# Optimised spectral effects of programmable LED arrays (PLA)s on bioelectricity generation from algal-biophotovoltaic devices

**DOI:** 10.1038/s41598-020-72823-9

**Published:** 2020-09-30

**Authors:** Fong-Lee Ng, Siew-Moi Phang, Boon Leong Lan, Vineetha Kalavally, Cheng-Han Thong, Kian-Ted Chong, Vengadesh Periasamy, Karthikeyan Chandrasekaran, G. Gnana kumar, Kamran Yunus, Adrian C. Fisher

**Affiliations:** 1 Institute of Ocean and Earth Sciences (IOES), University of Malaya, Kuala Lumpur, Malaysia; 2 Faculty of Applied Sciences, UCSI University, Kuala Lumpur, Malaysia; 3 Electrical and Computer Systems Engineering and Advanced Engineering Platform, School of Engineering, Monash University, Sunway, Malaysia; 4 Institute for Advanced Studies, University of Malaya, Kuala Lumpur, Malaysia; 5 Low Dimensional Materials Research Centre (LDMRC), Department of Physics, University of Malaya, Kuala Lumpur, Malaysia; 6 Department of Physical Chemistry, School of Chemistry, Madurai Kamaraj University, Madurai, Tamil Nadu 625021 India; 7 Department of Chemical Engineering and Biotechnology, University of Cambridge, Philipa Fawcett Drive, Cambridge, CB3 0AS UK

**Keywords:** Biotechnology, Biophysics

## Abstract

The biophotovoltaic cell (BPV) is deemed to be a potent green energy device as it demonstrates the generation of renewable energy from microalgae; however, inadequate electron generation from microalgae is a significant impediment for functional employment of these cells. The photosynthetic process is not only affected by the temperature, CO_2_ concentration and light intensity but also the spectrum of light. Thus, a detailed understanding of the influences of light spectrum is essential. Accordingly, we developed spectrally optimized light using programmable LED arrays (PLA)s to study the effect on algae growth and bioelectricity generation. *Chlorella* is a green microalga and contains chlorophyll*-a* (chl*-a*), which is the major light harvesting pigment that absorbs light in the blue and red spectrum. In this study, *Chlorella* is grown under a PLA which can optimally simulate the absorption spectrum of the pigments in *Chlorella*. This experiment investigated the growth, photosynthetic performance and bioelectricity generation of *Chlorella* when exposed to an optimally-tuned light spectrum. The algal BPV performed better under PLA with a peak power output of 0.581 mW m^−2^ for immobilized BPV device on day 8, which is an increase of 188% compared to operation under a conventional white LED light source. The photosynthetic performance, as measured using pulse amplitude modulation (PAM) fluorometry, showed that the optimized spectrum from the PLA gave an increase of 72% in the rETRmax value (190.5 μmol electrons m^−2^ s^−1^), compared with the conventional white light source. Highest algal biomass (1100 mg L^−1^) was achieved in the immobilized system on day eight, which translates to a carbon fixation of 550 mg carbon L^−1^. When artificial light is used for the BPV system, it should be optimized with the light spectrum and intensity best suited to the absorption capability of the pigments in the cells. Optimum artificial light source with algal BPV device can be integrated into a power management system for low power application (eg. environment sensor for indoor agriculture system).

## Introduction

Algae are being used in the development of biophotovoltaic devices (BPVs) for bioelectricity generation^1^. During photosynthesis, radiant energy absorbed by the algal cell drives the splitting of water to release a pair of electrons, which can be transported to the anode in the BPV device, and then to the cathode via an external circuit^2^. Ng et al.^3^ proved the formation of charge-transfer complex between algal biofilms, anode electrode and cathode electrode by measuring surface potential energy of different electrodes using the Kelvin probe method. Smaller gap in surface potential, higher electron donor/acceptor capacity and lower electrical resistivity of electrode contributed to higher power generation. Electron transfer can be direct, through endogenous mediators (eg. flavin), or through exogenous mediators (eg. polypyrrole), but direct electron transfer is preferable due to economy of cost and effort^4^. TiO_2_ nanowires^5^ and trimetallic Pt based (PtAuCu) alloy nanowires^6^, which exhibit significant electrocatalytic performance, high specific surface area, good biocompatibility, are able to improve fuel cell’s performance. Besides, nano-composite catalyst showed promising results: Kodali et al.^7^ enhanced the MFC performance by using an iron-graphence catalyst and Karthikeyan et al.^8^ reported 3D flower-like FeWO_4_/CeO_2_ on rGO produced high power and stable power output from alga BPV devices. BPV devices have been reported to generate current and power densities, respectively, in the range of 0.17 to 993 mA m^−2^ and up to 400 mW m^−2^ with various BPV reactors and operating parameters^1^. The maximum power density reported thus far was generated from a two-chamber device using an algae suspension, mediated electron transfer, and a gold-inscribed Nafion membrane^9^. However, the selection of algal strains with high photosynthetic performance, and providing these living energy-conversion microbes with the most suitable physiological conditions to perform their activities optimally is very important. Studies of improvised algal BPV systems were carried out to increase the power output from algal BPV devices^10–12^. However, the algal BPV has another important advantage over the MFC: while the MFC releases CO_2_, the algal BPV device consumes CO_2_ and releases O_2_. Therefore, the carbon negative algal BPV device represents a sustainable, environment-friendly technology amenable to low-power applications.

Irradiance, characterized by both quality and quantity, is a critical factor influencing photosynthetic performance^13^. Algal cells use a fraction (0.1%) of the radiant energy that is absorbed by chl*-a* in the chloroplast, for photosynthesis. Excess energy is dissipated as fluorescence and heat^14^, accompanied by a defense mechanism that triggers production of reactive oxygen species (ROS) to prevent photo-oxidative damage to the Photosystem II (PS II) in a process known as photoinhibition, which ultimately prevents cellular death^15^. In dense cultures, while photoinhibition may occur at the upper layer, cells in the lower layers may be light-deprived due to light attenuation as it penetrates the culture^15^, resulting in reduced biomass production^16^. Continuous exposure to high irradiance further damages the PS II apparatus which leads to buildup of these impaired units and failure in maintaining charge separation within the photosystem^17^.

A considerable amount of literature has been published on the effect of radiant spectrum and energy on photosynthesis, pigmentation and algal biomass production. Plants including algae use visible light, ranging from 400 to 700 nm, known as the photosynthetically active radiation (PAR) region, which are wavelengths with sufficient energy to support the photosynthetic process^18^. However, blue (420–450 nm) and red (660–700 nm) light are known to be as efficient for photosynthesis as the full spectrum. Chlorophylls, carotenoids and phycobilins comprise the pigments found in algae. Chlorophyl*-a* (chl*-a*) is the main pigment responsible for energy absorption while chlorophyll *b*, *c* and carotenoids are accessory pigments, with the latter performing photoprotection roles as well. The composition of pigments in algae defines the irradiance spectrum best suited for its photosynthetic performance. This is especially relevant when artificial light is used for cultivation. The Chlorophytes, or green algae, with chlorophylls being the dominant pigments, absorb mainly blue and red light. Chlorophytes are able to utilize blue light more efficiently than cyanobacteria, due to the loss of chl*-b* in cyanobacterial species^19^.

Photosynthesis in microalgae can be driven by sunlight or artificial light. Although sunlight is the most cost-effective energy source for microalgae production, consistent artificial lighting is still economically feasible when biomass is used as a feedstock for high value product such as food supplements^20^. LED light is an emerging and economical technology compared to ordinary fluorescent lamps in microalgal production because of its lower power consumption, longer life time, lower heat dissipation, smaller mass and volume, generation of lower heat when supplying light, longer life-span compared to fluorescence lamp, and higher conversion efficiency^21^. Green LEDs (525–550 nm) were often found to be unsuitable for microalgae culture if used without additional light source. Mixing of red and blue photons (red LEDs supplemented with 10 to 30% blue light) has often increased biomass production compared to red light alone^20^.

Intensive research efforts have been focused on how to achieve maximum growth of microalgae. One of the promising strategies is to provide a light source in which the irradiance spectrum is specifically tailored to the absorption spectrum of the light harvesting pigments. Sforza et al.^22^ reported improved growth rate of *Nannochloropsis salina* in photobioreactor using a white LED with a spectrum ranging from 400 to 780 nm. Maximum growth rate occurred with 150 µmol photons m^−2^ s^−1^ at 0.521 day^−1^^22^. Das et al.^23^ reported that *Nanochloropsis* sp. registered highest specific growth rate at 0.64 day^−1^ under blue light^23^. Mohsenpour and Willoughby^24^ presented growth of *Chlorella vulgaris* (Chlorophyte) and *Gloeothece membranacea* (Cyanophyte) under different light conditions. Results indicated that red light enhanced biomass production for both the strains, 0.135 gL^−1^ day^−1^ and 0.184 gL^−1^ day^−1^ for *Chlorella vulgaris* and *Gloeothece membranacea* respectively. Green light increased chlorophyll production in *Chlorella vulgaris* with an increase of 1.98% g/g cell)^25^. However, there is a lack of research concerning the effect of improved light quality on bioelectricity generation. In this study, the absorption spectrum of the chlorophyte *Chlorella* UMACC 313 was determined and used as the reference to provide irradiance through the programmable LED arrays (PLA)s. *Chlorella* sp. was investigated under two types of irradiance; the PLA based on the absorption spectrum of the *Chlorella* (treatment), and white LED light (control) to study the effectiveness of light quality on biomass production, photosynthetic performance, and bioelectricity generation.

## Results and discussion

BPV devices, containing either immobilized algae or suspended algae, were exposed to conventional white LED light, at 90 µmol photons m^−2^ s^−1^, and 25 ± 2 °C as a control. The treatment was exposure to light from the PLA using the simulated absorption spectrum of the *Chlorella* pigments. Light exposure was provided on a 12 h:12 h light:dark cycle in an incubator maintained at 25 ± 2 °C. Each condition was repeated in triplicate. Cultures were grown for 12 days, with measurements for growth, photosynthetic performance and power output taken on days 0, 4, 8, and 12. Power output measurements were taken during light and dark conditions, to determine if current (dark current) is also generated in the dark^11^.

### Absorption spectrum of chlorophylls and carotenoids from Chlorella UMACC 313

**Figure 1:**
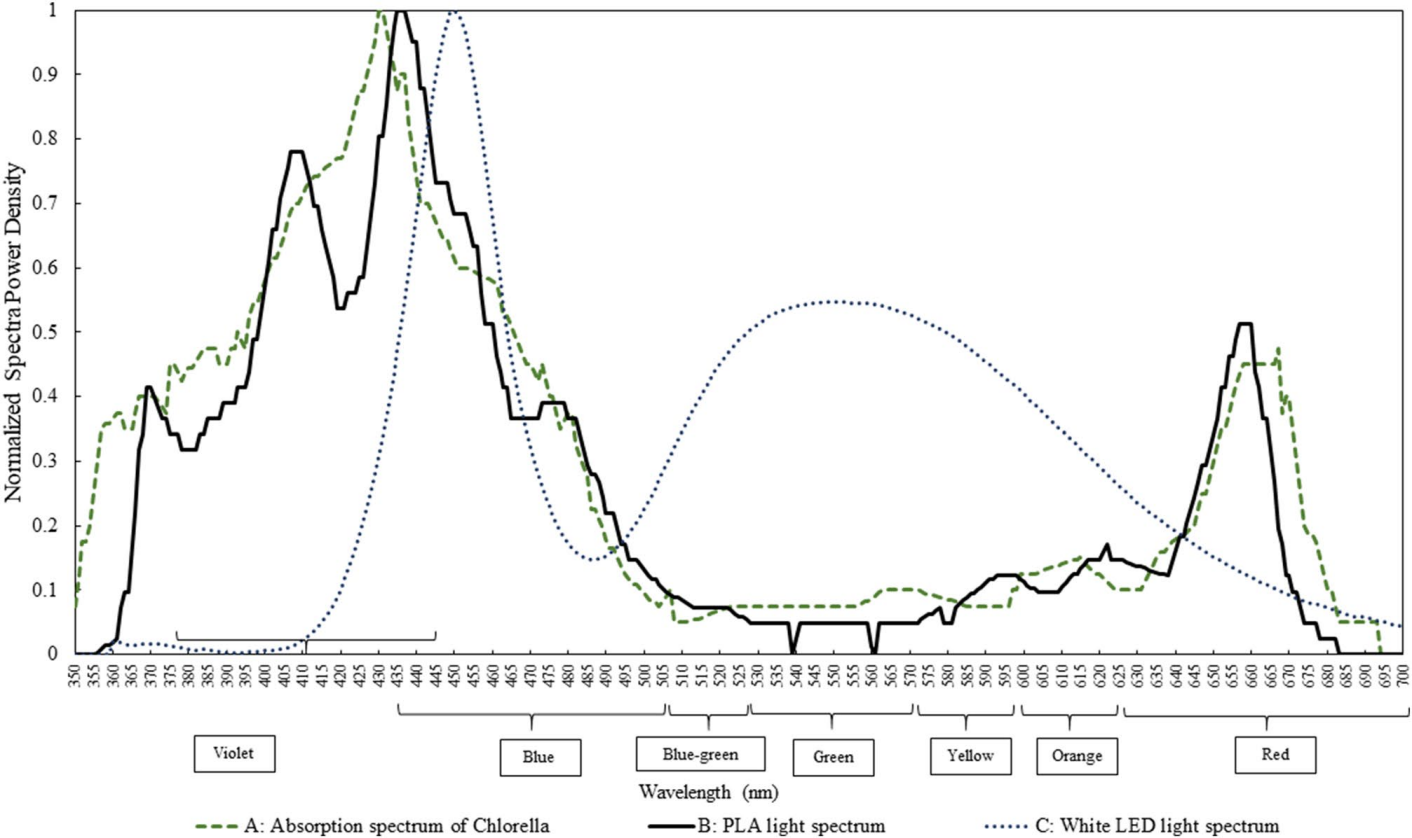
Comparison of three different spectra. (A) Measured absorption spectrum of *Chlorella* UMACC 313 (based on the extracted chl*-a* and carotenoid pigments), (B) simulated absorption spectrum of *Chlorella* UMACC 313 produced by the PLA, (C) spectrum of conventional LED white light.

The measured absorption spectrum of the pigments (chlorophylls and carotenoids) from *Chlorella* UMACC 313 is compared with the spectrum of the white LED light, and the simulated absorption spectrum of the *Chlorella* pigments generated by the PLA (Fig. 1).

There was good compatibility between the measured and simulated absorption spectrum of the pigments. Emission spectrum of simulated illumination produced by the PLA corresponded very closely to absorption spectrum of *Chlorella* UMACC 313, including the spectral region absorbed by the chl*-a* and carotenoid pigments (wavelength peaks at 370, 410, 435 and 660 nm). Although the white light spectrum, with wavelength peaks at 450 and 560 nm, did not compare very well with the other two spectra, there was sufficient overlap with the measured absorption spectrum of *Chlorella* UMACC 313 to allow use of white light for algal culture.

### Growth of Chlorella UMACC 313 in the algal BPV devices

Table 1 gives details of the cell density along with chl*-a* and carotenoid content of the algae grown in the BPV with immobilized and suspended algae, under control (exposure to conventional white LED light) and treatment (exposure to light from the PLA) conditions on days 0, 4, 8, and 12. The specific growth rates, µ, based on chl*-a* and calculated from day 4 to day 8 (exponential phase) of the cultures, were 0.57 day^−1^ and 0.85 day^−1^ under control condition, and 0.82 day^−1^ and 0.92 day^−1^ under treatment conditions for the suspended and immobilized cultures, respectively. Growth rate and cells density of microalgae were higher in immobilized cultures. In alginate, carboxylic groups are naturally present and randomly distributed, and some monomeric units may have more than one carboxylic group^26^. This may contribute to better contact between the microalgae cells and ITO anode in this study. The immobilized algae grow faster when immobilized inside the harmless and non-toxic alginate gel, where nutrients from the medium freely diffuse through the porous gel. Our data showing slower growth of cells in the immobilized condition from day 0 to day 4 supports an interpretation that it took time for these cells to acclimatize to the physical change of environmental conditions compared to suspended cells^11^. However, once the cells had adapted to being encapsulated within the alginate gel matrix, cell density increased rapidly for the remainder of the experiment, from day 4 through day 12.

**Table 1:** Statistical comparison of cell density (× 10^7^ cells mL^−1^), chl*-a* concentration (mg L^−1^) and carotenoid concentration (mg L^−1^) in algal-biophotovoltaic device with suspended and immobilized of *Chlorella* UMACC 313 under conventional white LED light or PLA on days 0, 4, 8, and 12. Data as means ± S.D. (n = 3).

Light source	Day	Culture condition	Cell density (× 10^7^ cells mL^−1^)	Chl*-a* (mg L^−1^)	Carotenoids (mg L^−1^)
Conventional white LED light 90 µmol photons m^−2^ s^−1^	0	Suspended	3.67 ± 0.76^cd^	0.129 ± 0.008^f^	0.073 ± 0.010^e^
		Immobilized	5.17 ± 0.29^cd^	0.362 ± 0.040^f^	0.150 ± 0.020^de^
	4	Suspended	9.5 ± 1.8^cd^	1.28 ± 0.14^e^	0.523 ± 0.068^de^
		Immobilized	17 ± 1^cd^	10.80 ± 0.33^b^	5.781 ± 0.002^b^
	8	Suspended	38.3 ± 3.8^bc^	4.53 ± 0.60^d^	1.58 ± 0.15^d^
		Immobilized	98 ± 5^b^	13.23 ± 0.34^b^	8.1 ± 1.0^a^
	12	Suspended	50. ± 5^b^	2.89 ± 0.18^e^	1.03 ± 0.16^de^
		Immobilized	145 ± 19^a^	9.79 ± 0.72^c^	4.82 ± 0.24^bc^
PLA 90 µmol photons m^−2^ s^−1^	0	Suspended	2.17 ± 0.29^d^	0.141 ± 0.012^f^	0.100 ± 0.009^e^
		Immobilized	3.5 ± 0.5^cd^	0.390 ± 0.045^f^	0.223 ± 0.027^de^
	4	Suspended	12.5 ± 2.5^cd^	3.72 ± 0.25^de^	1.100 ± 0.071^d^
		Immobilized	10.3 ± 1.5^cd^	15.52 ± 0.45^a^	6.37 ± 0.47^ab^
	8	Suspended	26.7 ± 3.8^c^	7.54 ± 0.42^c^	2.69 ± 0.23^c^
		Immobilized	223 ± 19^a^	16.42 ± 0.23^a^	7.00 ± 0.13^a^
	12	Suspended	53.3 ± 7.6^b^	5.41 ± 0.11^d^	1.97 ± 0.19^ cd^
		Immobilized	164.2 ± 6.7^a^	7.22 ± 0.16^c^	4.69 ± 0.39^c^

Different letters indicate significant different between different value (ANOVA, Tukey̕ s HSD test, p < 0.05).

Microalgae reproduction capacity depends on the characteristic of the light wavelength^27^. In this study, higher growth rate was observed when the algal cells were grown in the simulated spectrum that was close to the absorption spectrum of the pigments of *Chlorella* UMACC 313. Specifically, when the BPVs were exposed to treatment conditions, growth rates determined based on chl*-a* increased by 43.9% for suspended and 12.2% for immobilized cultures compared to the control condition. The lower percent increase in immobilized cultures might be due to an increase in cell density within the alginate film that caused self-shading and/or a decrease in nutrient diffusion efficiency that limited cell growth and led to lower power output.

Generally, pigment contents (chl*-a* and carotenoid) in immobilized algae were observed to be higher compared to suspended algae cultures. Higher chl*-a* content occurred on day 4 (15.52 mg L^−1^) and day 8 (16.42 mg L^−1^) compared to day 0 (0.390 mg L^−1^) and day 12 (7.22 mg L^−1^) under treatment conditions. Higher chl*-a* content in immobilized state on day 4 and day 8 may be due to the high irradiance to the thin layer of gel film that led to high physiological activities of the algal cells. However chl*-a* content decreased on day 12 (7.22 mg L^−1^), which may be due to nutrient limitation and CO_2_ diffusion limitation occurring in the immobilized cells because of high cell density inside the alginate films and relative to lower power density (0.332 mW m^−2^) compared to day 8 (0.451 mW m^−2^)^28^.

The highest carotenoid content was observed on day 8 (8.1 mg L^−1^) when the immobilized *Chlorella* cells were exposed to control conditions, followed by *Chlorella* cells in immobilized state exposed to treatment conditions (7.00 mg L^−1^). This high amount of carotenoid content may represent a protective response to environmental stress and low photosynthetic efficiency of the cells (α = 0.558). In this study, high cell density and limited nutrients were observed on day 8 and likely caused algae cells to accumulate carotenoids to preserve cells from oxidative damage by reducing photosynthetic activity metabolic rate. Reduction of photosynthetic electrons transport may be associated with the formation of free radicals and singlet oxygen. In order to prevent damage from photooxidation, microalgae are able to synthesize a large amount of carotenoids, which can deactivate these dangerous oxidant molecules^29^.

Carbon fixation in the algal BPV devices was computed on day 8. Under the control light source, carbon fixation by suspended and immobilized algal cells, was 150 and 440 mg carbon L^−1^, respectively. Under the treatment light source, carbon fixation by suspended and immobilized algal cells was 250 and 550 mg carbon L^−1^, respectively. Under the control light source, CO_2_ fixation rate was found to be 57 and 123 mg CO_2_ L^−1^ day^−1^ for the suspended and immobilized algae, respectively. Under the treatment light source, CO_2_ fixation rate was 68 and 198 mg CO_2_ L^−1^ day^−1^ for the suspended and immobilized algae, respectively. During photosynthesis, CO_2_ is utilized in the Calvin–Benson Cycle through regulation of the enzyme ribulose bisphosphate carboxylase–oxygenase (Rubisco); a declining Rubisco level induces higher CO_2_ uptake. The enzyme carbonic anhydrase (CA) will respond to different conditions. If necessary, it will convert bicarbonate, HCO_3_^−^, to CO_2_ for Rubisco-driven metabolic activities. Hence, the regulation of Rubisco activity is dependent on light source and directly affects the carbon fixation rate by microalgae^30,31^. This explains why carbon and CO_2_ fixation rates were higher under the treatment light source for both suspended and immobilized cultures. Under the treatment light source, *Chlorella* cells showed better photosynthetic performances, with high Alpha and rETRmax values and improved efficiency of the carbon fixation process. All these values are based on small BPV devices; for future work, modules of the device will be stacked to increase the power output.

Temperature, pH, nutrients and light quality (wavelength) are responsible for intensifying the CO_2_-conversion in algal cells. Captured carbon from atmosphere is pumped by bicarbonate transporters present in plasma membrane and chloroplast in algal cells. Bicarbonate is converted into CO_2_ that can be fixed by rubisco (ribulose bisphosphate carboxylase oxygenase) inside the chloroplast to produce two molecules of 3-phosphoglycerate. Three carbon organic acids are then reduced to the sugars for starch and lipid production. Oxygen can compete with CO_2_ for fixation by rubisco and therefore reduce the CO_2_-fixation rate. To overcome this, alga cells pump sufficient bicarbonate into cells to ensure the CO_2_ concentration levels are higher than those achievable by equilibrium with air^32^.

Impact analysis of our algal biophotovoltaic devices was conducted using the OpenLCA software (Version 1.10.3). In our model, we set five algal BPV devices as basis and identified to our best ability, the resources required in producing our devices so that relevant calculations can be made by the software. We note that the data generated are estimated values, as they may differ depending on the databases used for the input section in our model graph. The impact assessment method that we used was CML2001—Apr. 2013. We found that the estimated amount of carbon dioxide emitted to the atmosphere as a result of developing our algal BPV device system (in replicates of five) is approximately 0.308 kg CO_2_. The main contributing factors are the polymethyl methacrylate (PMMA) used in manufacturing the cylindrical chamber of the algal BPV device and the copper wires used to complete the circuit. This system also leads to an abiotic depletion (ADP elements) of 2.15 × 10^–5^ kg Sb eq.

### Photosynthetic performance of Chlorella UMACC 313 in the algal BPV devices

Table 2 gives details of the photosynthetic performance of *Chlorella* in the BPVs, under control and treatment conditions, on days 0, 4, 8, and 12. Fluorescence characteristics of the BPV devices exposed to different light source were different, indicating a strong effect of the light spectrum on photosynthetic performance.

**Table 2:** Statistical comparison of photosynthetic performance of algal-biophotovoltaic device with suspended and immobilized of *Chlorella* UMACC 313 under conventional white LED light and PLA on days 0, 4, 8, and 12. Data as means ± S.D. (n = 3).

Light source	Day	Culture condition	Fv/Fm	Alpha (α)	rETRmax (µmol electrons m^−2^ s^−1^)	Ek (µmol photons m^−2^ s^−1^)
Conventional White LED Light 90 µ mol photons m^-2^ s^-1^	0	Suspended	0.226 ± 0.018^ef^	0.246 ± 0.042^g^	39.7 ± 3.3^gh^	164 ± 30^cde^
		Immobilized	0.317 ± 0.042^ef^	0.422 ± 0.015^ef^	24.2 ± 3.7^h^	57.2 ± 7.4^h^
	4	Suspended	0.476 ± 0.055^ed^	0.539 ± 0.041^cde^	69.1 ± 1.3^def^	129 ± 12^efg^
		Immobilized	0.517 ± 0.022^bc^	0.596 ± 0.048^cd^	58.3 ± 3.5^defg^	99 ± 13^gh^
	8	Suspended	0.693 ± 0.013^a^	0.559 ± 0.048^cde^	125.2 ± 6.9^c^	224.3 ± 6.8^bc^
		Immobilized	0.647 ± 0.066^ab^	0.558 ± 0.028^cde^	110.5 ± 3.9^c^	198 ± 15^bcd^
	12	Suspended	0.705 ± 0.016^a^	0.614 ± 0.055^c^	52.11 ± 0.26^fg^	85.2 ± 7.1^gh^
		Immobilized	0.697 ± 0.026^a^	0.552 ± 0.015^cde^	56.4 ± 4.2^efg^	102.1 ± 8.0^fgh^
PLA 90 µ mol photons m^−2^ s^−1^	0	Suspended	0.196 ± 0.023^f^	0.358 ± 0.014^fg^	38.368 ± 0.010^gh^	107.4 ± 4.1^efgh^
		Immobilized	0.348 ± 0.005^de^	0.451 ± 0.014^def^	41.4 ± 2.8^gh^	91.8 ± 5.1^gh^
	4	Suspended	0.523 ± 0.067^bc^	0.599 ± 0.060^cd^	254 ± 15^a^	427 ± 54^a^
		Immobilized	0.516 ± 0.061^bc^	0.372 ± 0.065^fg^	76 ± 15^de^	203.2 ± 4.8^bcd^
	8	Suspended	0.601 ± 0.045^abc^	0.794 ± 0.069^ab^	126 ± 18^c^	160 ± 30^def^
		Immobilized	0.631 ± 0.011^ab^	0.844 ± 0.090^a^	190.5 ± 5.4^b^	277 ± 20^b^
	12	Suspended	0.589 ± 0.035^abc^	0.618 ± 0.079^c^	130.1 ± 6.9^c^	212 ± 21^bcd^
		Immobilized	0.611 ± 0.025^abc^	0.642 ± 0.054^bc^	81.8 ± 4.4^d^	127.7 ± 5.2^efg^

Different letters indicate significant difference between different values (ANOVA, Tukey̕ s HSD test, p < 0.05).

The maximum quantum yield (F_v_/F_m_) which is used to indicate physiological health in the algae is inversely related to stress levels of the cells. The threshold for stress in *Chlorella* is Fv/Fm = 0.5^10^. Low F_v_/F_m_ values were observed on day 0 for suspended and immobilized *Chlorella* culture under both conditions. This is due to adjustment of the inoculum in a new environment. Fv/Fm values of the suspended algae was lower (p < 0.05) under treatment conditions compared to the control, and this trend continued throughout the experiment. Increased Fv/Fm values may be due to higher photosynthetic activity under optimised irradiance. As the cultures grew, the physiological health of the *Chlorella* cells improved, and higher F_v_/F_m_ values (> 0.45, p < 0.05) were observed starting on day 4 for all conditions. Day 4 to day 8 is considered to be the exponential growth phase.

Photosynthetic efficiency (PE) alpha (α) is an important indicator to define the fraction of available incident light within the solar spectrum that is stored as chemical energy in biomass. Increasing PE is a promising approach to enhance microalgae productivity. PE is derived as the fraction of light energy converted into chemical energy during photosynthesis process^33^. α values increased from day 0 to day 8, as the algae adjusted and grew. There was a small reduction in α on day 12, especially in the immobilized cells. This may be due to reduced light availability per cell due to higher cell density. The algae in the BPVs exposed to treatment condition had higher α (p < 0.05) compared to the control. On day 8, α = 0.794 and 0.844 for suspended and immobilized algae, respectively. Cells exposed to the simulated spectrum (treatment) showed higher α values, presumably because the spectrum was closer to the absorption spectrum of the *Chlorella* cells, allowing the pigments to more efficiently use the energy for photosynthesis.

The maximum relative electron transport rate (rETR_max_) was higher in the algae exposed to treatment condition, with the highest value on day 4 (254 µmol electrons m^−2^ s^−1^) for suspended algae and day 8 (190.5 µmol electrons m^−2^ s^−1^) for immobilized algae. The higher rETR_max_ occurred during the exponential growth phase, when the cells were actively growing, and biomass was highest (Table 1). The relationship between rETR_max_ and power output will be discussed in the next section.

The photoadaptive index (E_k_) was significantly higher (p < 0.05) under treatment condition and ranged from 91.8 µmol photons m^−2^ s^−1^ (day 0, immobilized algae) to 427 µmol photons m^−2^ s^−1^ (day 4, suspended algae). In general, the adaptive index was higher in suspended algae compared to immobilized algae. In the BPV with suspended algae, the cells were dispersed in the medium, compared to the immobilized culture where the cells were found only in the approximately 2 mm thick alginate film. Thus it appears that the highly dispersed condition allowed for a higher light saturation level^11^.

Autotrophic microalgae use visible light (400–700 nm) as the main source of radiant energy to generate adenosine triphosphate (ATP) and nicotinamide adenine dinucleotide phosphate (NADPH) via the photosynthesis process^18^. The energy content of irradiance at wavelengths above 750 nm is too low to mediate a chemical change in microalgal cells, hence radiant energy absorbed in this range is dissipated as thermal energy^34^. Irradiance of 380 nm and below has ionizing effects. So, wavelengths between 380 and 750 nm have energy able to produce chemical change in the absorbing molecules, as happens throughout the photosynthetic pathway^18^. For *Chlorella* UMACC 313 used in this study, major wavelengths utilised by the pigments are within the range 420–440 nm and 650–670 nm, and accessory wavelengths are located below, between or above these ranges^20^. Light energy emitted by the PLA provides ideal energy levels required by *Chlorella* UMACC 313 to initiate photosynthesis, and the highest α and rETR_max_ were stimulated by the PLA in this experiment.

### Bioelectricity generation by Chlorella UMACC313 in the algal BPV devices

**Figure 2:**
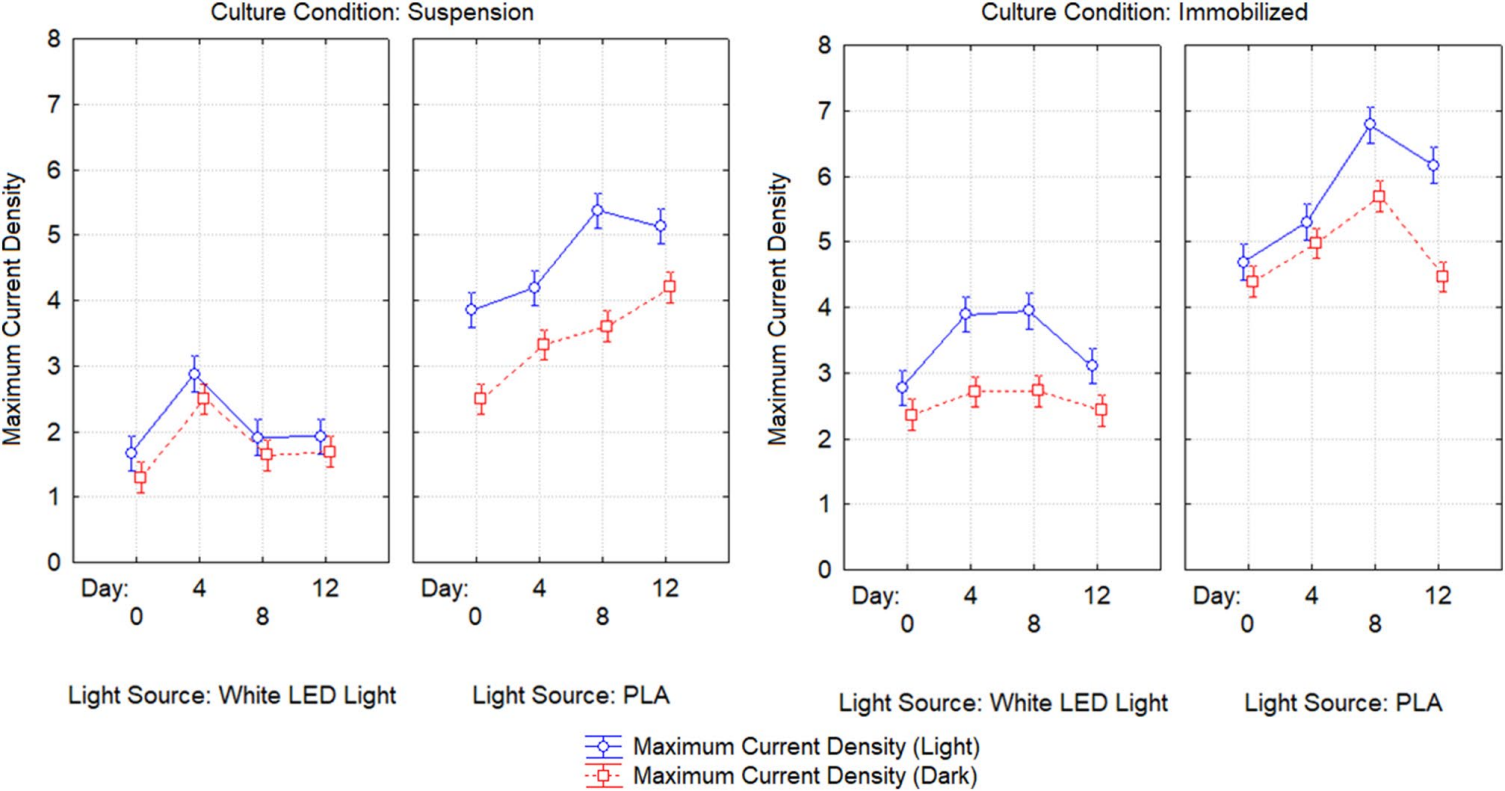
Maximum current density of algal-biophotovoltaic device with suspended and immobilized of *Chlorella* UMACC 313 in dark and light conditions under conventional white LED white light and PLA on days 0, 4, 8, and 12. Data as means ± S.D. (n = 3).

**Figure 3:**
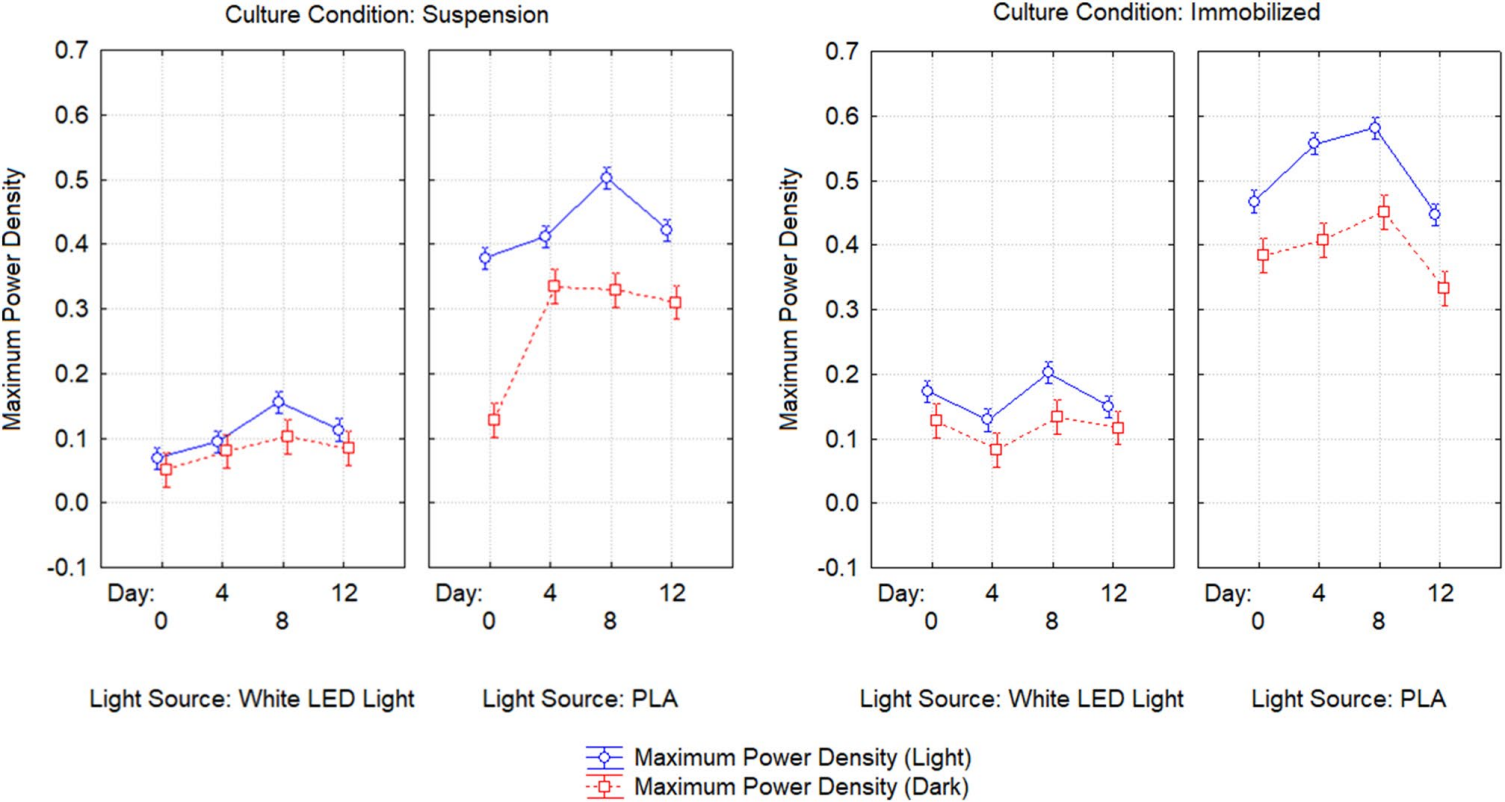
Maximum power density of algal-biophotovoltaic device with suspended and immobilized of *Chlorella* UMACC 313 in dark and light conditions under conventional white LED white light and PLA on days 0, 4, 8, and 12. Data as means ± S.D. (n = 3).

Table 3 shows the details of the power output from the BPVs containing suspended and immobilized algae, under control and treatment conditions, on days 0, 4, 8, and 12. Power density was measured under light and dark conditions. Figures 2 and 3 show maximum current density and maximum power density of algal-biophotovoltaic device with suspended and immobilized of *Chlorella* UMACC 313 in dark and light conditions under conventional white LED light and PLA on days 0, 4, 8, and 12. In a previous study, we reported that our alginate immobilized algal-BPV produced a peak power density (0.289 mW m^−2^) on day 8^11^. In the present study, the highest power density was recorded from immobilized algal-BPV on day 8 (exponential phase culture) for both light treatments. When the immobilized algal-BPV was exposed to control condition, the highest power densities were 0.202 mW m^−2^ and 0.133 mW m^−2^ in light and dark conditions, respectively. When the immobilized algal-BPV was exposed to treatment conditions, there was an increase of power density from 0.202 to 0.581 mW m^−2^ (65.2%) in the light, and from 0.133 to 0.451 mW m^−2^ (70.5%) in the dark. The reason for the high power output is the same as for higher photosynthetic performance; that is, the spectrum provided by the PLA was compatible with the absorption spectrum of the algal pigments^11^.

**Table 3:** Statistical comparison of power output of algal-biophotovoltaic device with suspended and immobilized of *Chlorella* UMACC 313 in dark and light conditions under conventional white LED light and PLA on days 0, 4, 8, and 12.

Day	Light source	Condition	Maximum current density (mA m^−2^)		Maximum power density (mW m^−2^)	
			Light	Dark	Light	Dark
0	Conventional white LED light 90 µmol photons m^−2^ s^−1^	Suspended	1.667 ± 0.024^f^	1.297 ± 0.034^h^	0.069 ± 0.010^j^	0.051 ± 0.004^e^
		Immobilized	2.769 ± 0.072^e^	2.363 ± 0.028^g^	0.173 ± 0.008^fg^	0.127 ± 0.011^d^
4		Suspended	2.87 ± 0.21^e^	2.495 ± 0.047^g^	0.095 ± 0.005^ij^	0.079 ± 0.001^de^
		Immobilized	3.889 ± 0.076^d^	2.714 ± 0.048^g^	0.129 ± 0.004^hi^	0.0824 ± 0.011^de^
8		Suspended	1.91 ± 0.12^f^	1.640 ± 0.072^h^	0.156 ± 0.008^gh^	0.102 ± 0.002^de^
		Immobilized	3.95 ± 0.10^d^	2.724 ± 0.077^fg^	0.202 ± 0.012^f^	0.133 ± 0.002^d^
12		Suspended	1.93 ± 0.11^f^	1.697 ± 0.091^h^	0.113 ± 0.006^hij^	0.084 ± 0.004^de^
		Immobilized	3.10 ± 0.38^e^	2.43 ± 0.27^g^	0.149 ± 0.014^gh^	0.117 ± 0.007^de^
0	PLA 90 µmol photons m^−2^ s^−1^	Suspended	3.85 ± 0.26^d^	2.493 ± 0.045^g^	0.378 ± 0.013^e^	0.128 ± 0.008^d^
		Immobilized	4.69 ± 0.10^bc^	4.40 ± 0.32^bc^	0.468 ± 0.004^bc^	0.384 ± 0.055^ab^
4		Suspended	4.19 ± 0.21^cd^	3.32 ± 0.21^ef^	0.418 ± 0.027^de^	0.334 ± 0.042^bc^
		Immobilized	5.30 ± 0.14^b^	4.977 ± 0.015^b^	0.557 ± 0.029^a^	0.408 ± 0.009^a^
8		Suspended	5.37 ± 0.17^b^	3.61 ± 0.23^de^	0.502 ± 0.026^b^	0.329 ± 0.026^bc^
		Immobilized	6.78 ± 0.30^a^	5.70 ± 0.37^a^	0.581 ± 0.009^a^	0.451 ± 0.034^a^
12		Suspended	5.13 ± 0.46^b^	4.20 ± 0.18^cd^	0.422 ± 0.009^de^	0.310 ± 0.028^c^
		Immobilized	6.17 ± 0.38^a^	4.47 ± 0.41^bc^	0.440 ± 0.009^cd^	0.332 ± 0.017^bc^

Data as means ± S.D. (n = 3). Different letters indicate significant difference between different values (ANOVA, Tukey̕ s HSD test, p < 0.05).

**Figure 4:**
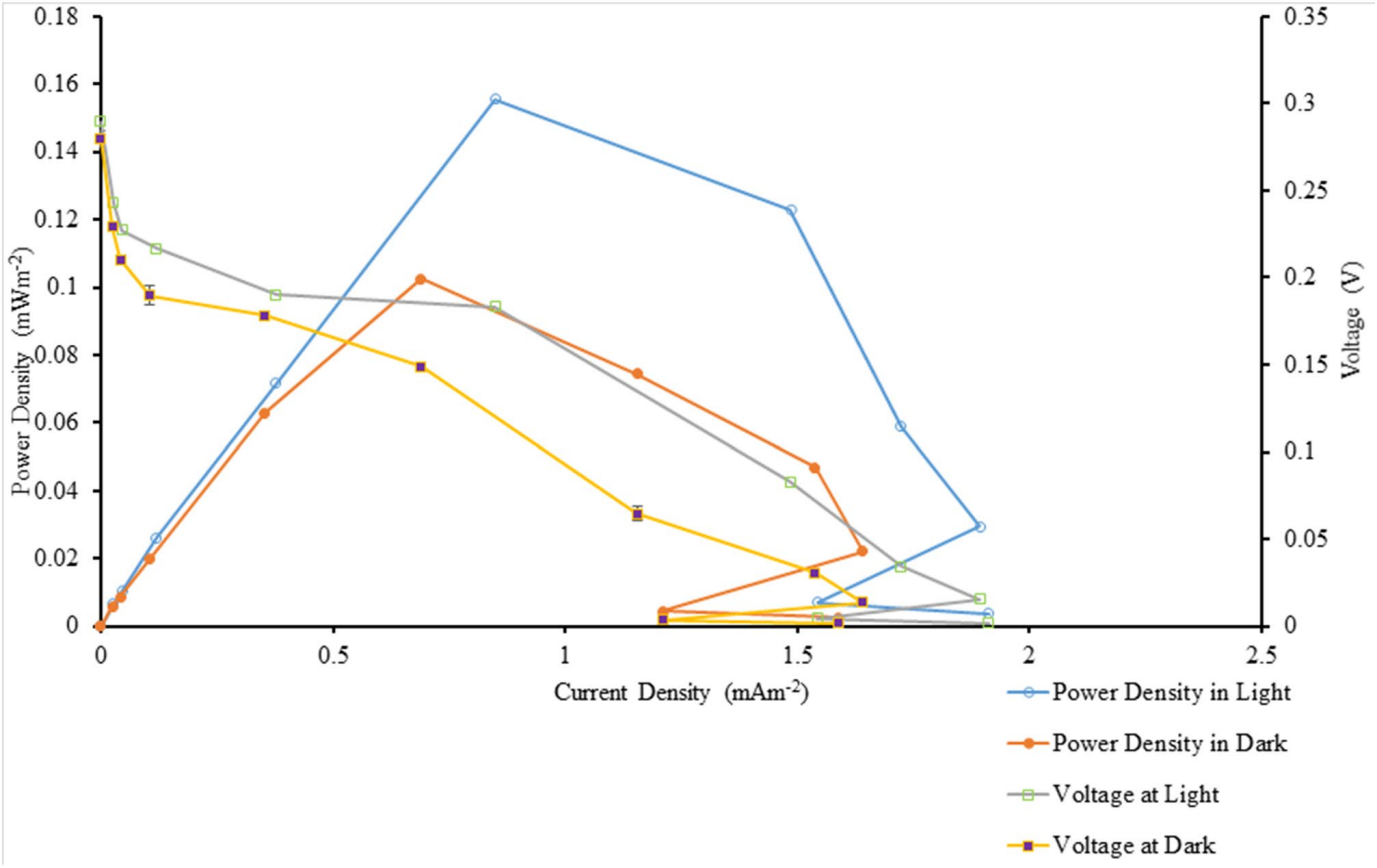
The polarization curve from suspended *Chlorella* UMACC 313 biophotovoltaic devices under conventional LED white light (90 µmol photons m^−2^ s^−1^) on day 8. Data as means ± S.D. (n = 3).

**Figure 5:**
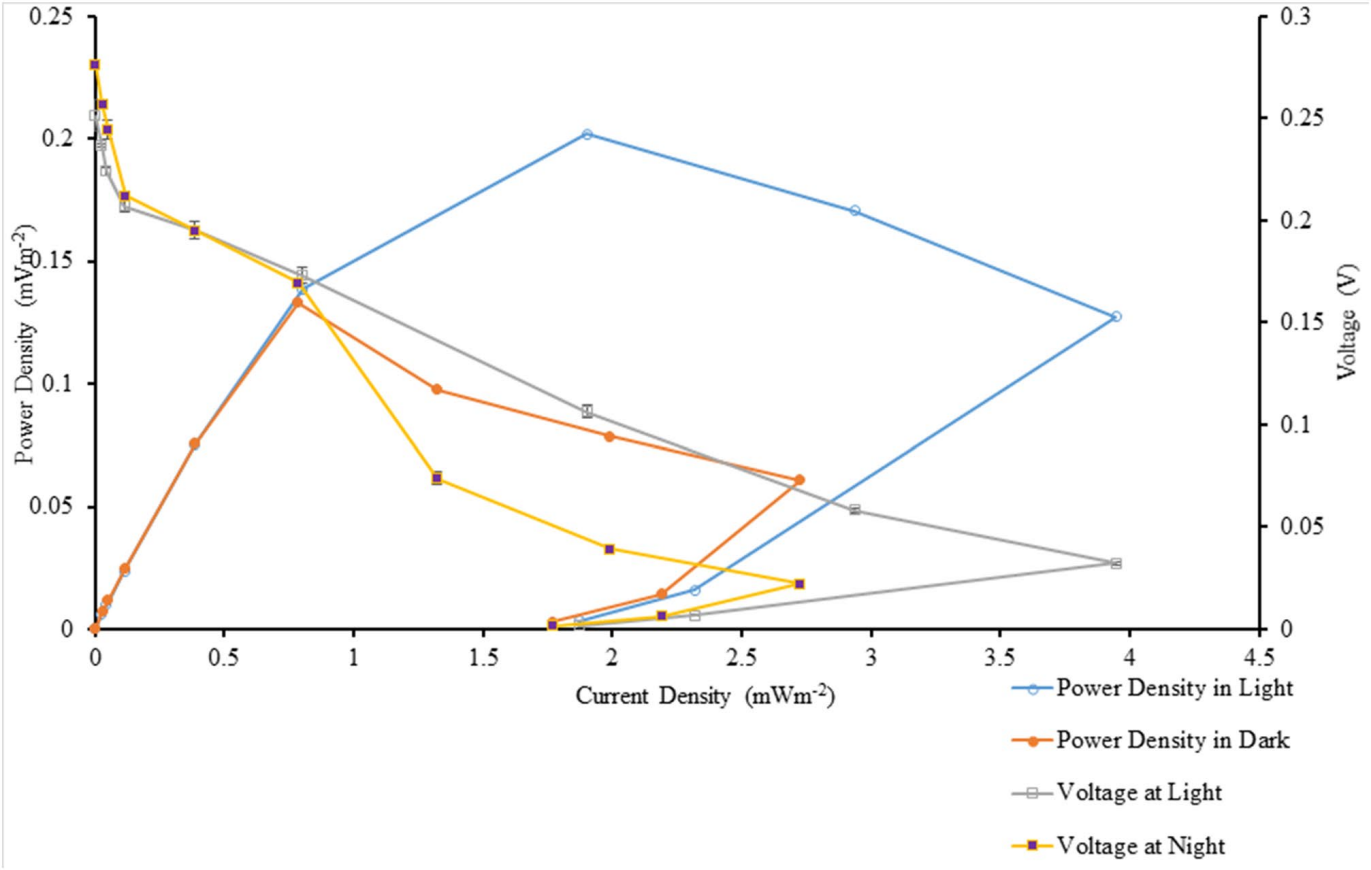
The polarization curve from immobilized *Chlorella* UMACC 313 biophotovoltaic devices under conventional LED white light (90 µmol photons m^−2^ s^−1^) on day 8. Data as means ± S.D. (n = 3).

**Figure 6:**
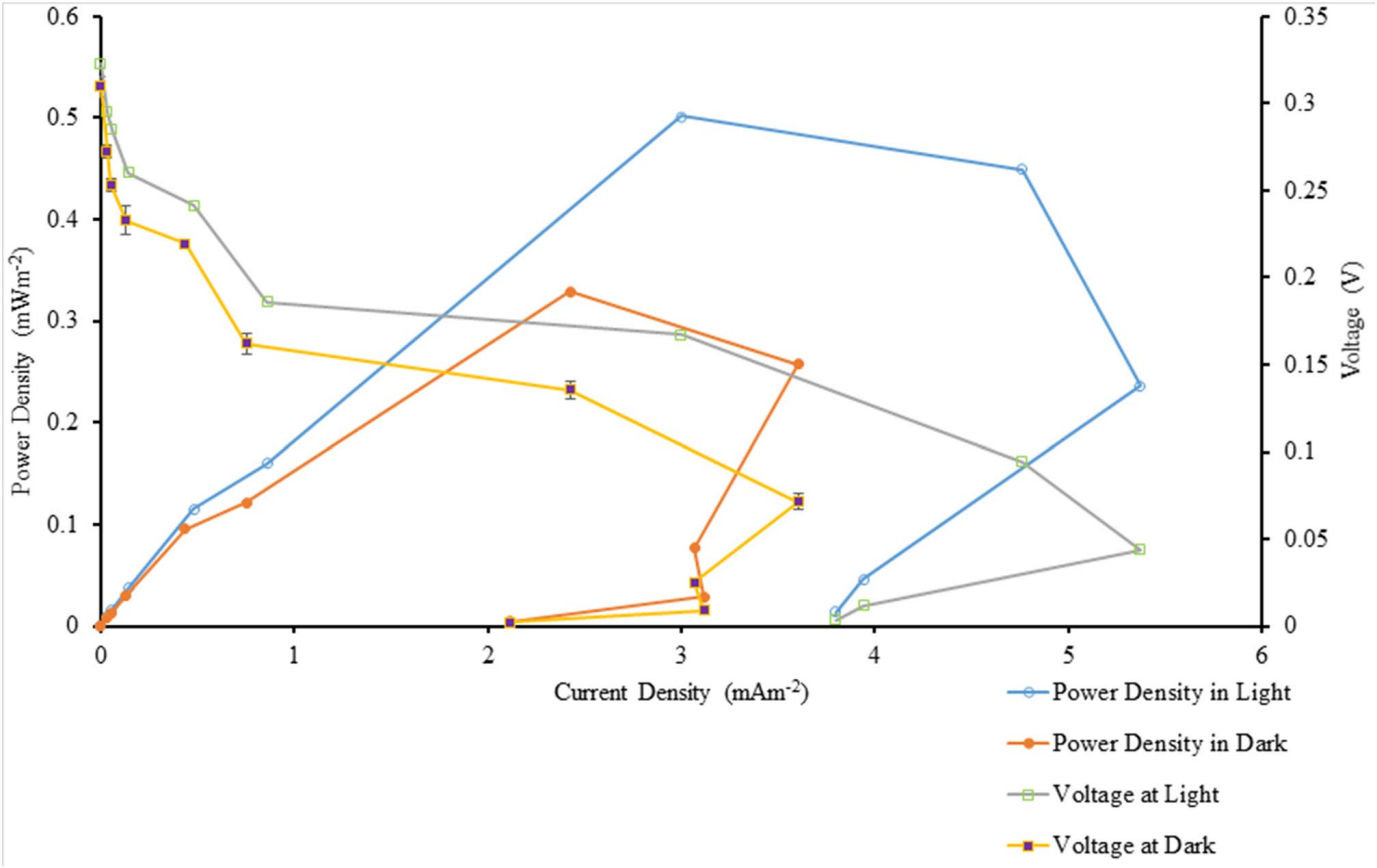
The polarization curve from suspended *Chlorella* UMACC 313 biophotovoltaic devices under PLA (90 µmol photons m^−2^ s^−1^) on day 8. Data as means ± S.D. (n = 3).

**Figure 7:**
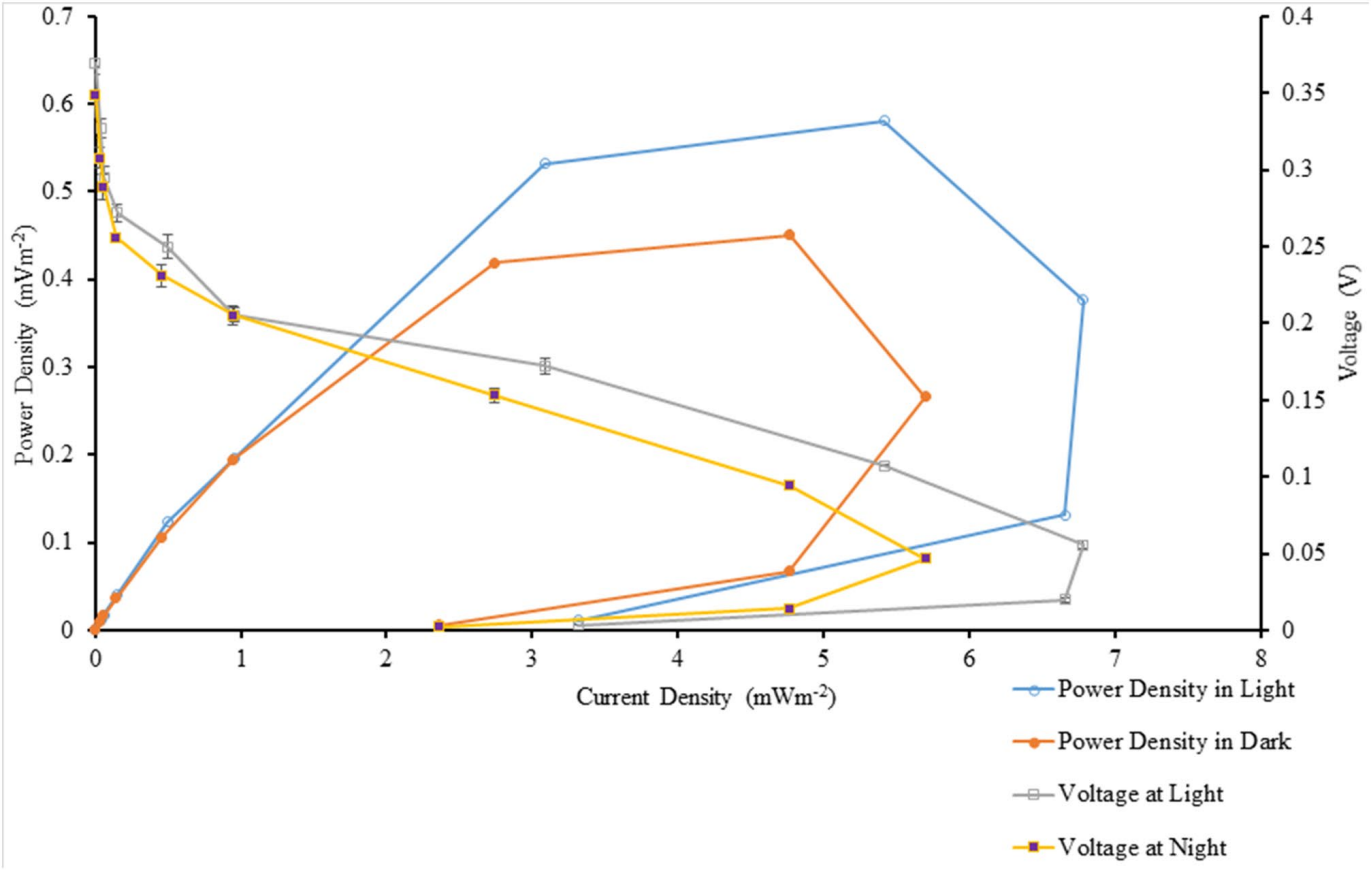
The polarization curve from immobilized *Chlorella* UMACC 313 biophotovoltaic devices under PLA (90 µmol photons m^−2^ s^−1^) on day 8. Data as means ± S.D. (n = 3).

In our present study, power output based on algal biomass (using chl*-a* content, Table 1) was estimated to be 5.09 mW m^−2^/mg chl*-a* (light condition) and 3.35 mW m^−2^/mg chl*-a* (dark condition) when the immobilized algal-BPV was exposed to control condition. When the immobilized algal-BPV was exposed to the treatment conditions, the power output based on algal biomass (using chl*-a* content) was higher, at 11.80 mW m^−2^/mg chl*-a* and 9.16 mW m^−2^/mg chl*-a*, respectively. Figures 4 and 5 show the polarization curves of suspended and immobilized algae BPV under control conditions, while Figs. 6 and 7 show polarization curves under treatment conditions.

The data in the present study indicates that the higher power output generated from the algal-BPV devices exposed to treatment conditions compared to control conditions may be attributed to optimum light quality received by *Chlorella* cells. Improved biomass production and photosynthetic performance led to improved bioelectric generation, with the use of optimized light spectrum. Microalgal PSII is enhanced by red light wavelengths while PSI is enhanced by blue light wavelengths; blue light enhances chlorophyll and carotenoid synthesis, as well as nitrogen metabolism^35^. Higher light intensity (90 μmol photons m^−2^ s^−1^) in the current study also contributed to the higher power output. Li et al.^36^ reported the effects of white LED light of different light intensities on *Chlorella pyrenoidosa*; the highest growth rate, 97.8 mg L^−1^ occurred at 90 μmol photons m^2^ s^−1^.

While the BBM provides complete nutrients for microalgal growth, ions in the medium also influence the bioelectricity generation from the algal BPV device. Electrical conductivity in liquid depends on the concentration of ions, such as hydrogen (H^+^), hydroxide (OH^−^), phosphate and nitrate, which are also nutrients present in BBM thus contribute to the conductivity^37^. In our previous study, we reported that power density of 0.066 mW m^−2^ was detected in our BPV device (only BBM medium and without microalgae). However, the power generated by the algae with BBM (0.345 ± 0.056 mW m^−2^) was significantly higher (ANOVA, P < 0.05), indicating that the *Chlorella* UMACC 313 cells were the main contributing factor in power generation from the algal BPV devices^38^.

## Conclusion

In photoautotrophic algal growth, radiant energy drives the metabolic activities of the cells, through production of chemical energy and reducing power via photosynthesis. In addition, a new role of the photosynthetic algal cells is the production of bioelectricity. Wavelength and intensity characteristics of the light source are critical factors for the growth of algae. This study has shown that when artificial light is provided, as through the PLA, a peak power output of 0.581 mW m^−2^ was generated, which is an increase of 188% compared to operation under a conventional white LED light source. For this study, we manipulated the light quality to provide spectrally optimised light for the pigments (specifically chl*-a*) of *Chlorella* UMACC 313 and demonstrated improved biomass production, photosynthetic performance and bioelectricity generation. Realization of a pilot scale energy harvesting system can be achieved when optimum artificial light source combined with algal BPV devices are integrated into a power management system for low power applications (eg. environment sensor for indoor agriculture system).

## Methods

### Algal culture

A local tropical algal strain from the University of Malaya Algae Culture Collection (UMACC) *Chlorella* UMACC 313, a chlorophyte, isolated from palm oil mill effluent, was used for this study. Stock cultures were prepared by inoculating exponential phase culture (20% inoculum) standardized at an optical density (OD) of 0.2 at 620 nm (OD_620nm_) into Bold’s Basal Medium (BBM)^39^ in 1 L conical flasks (total volume of culture: 500 mL). The flasks were placed in an incubator shaker (120 rpm) at 25 °C, with irradiance of 40 μmol photons m^−2^ s^−1^ on a 12:12 light:dark cycle.

### Preparation of algal BPV devices

**Figure 8:**
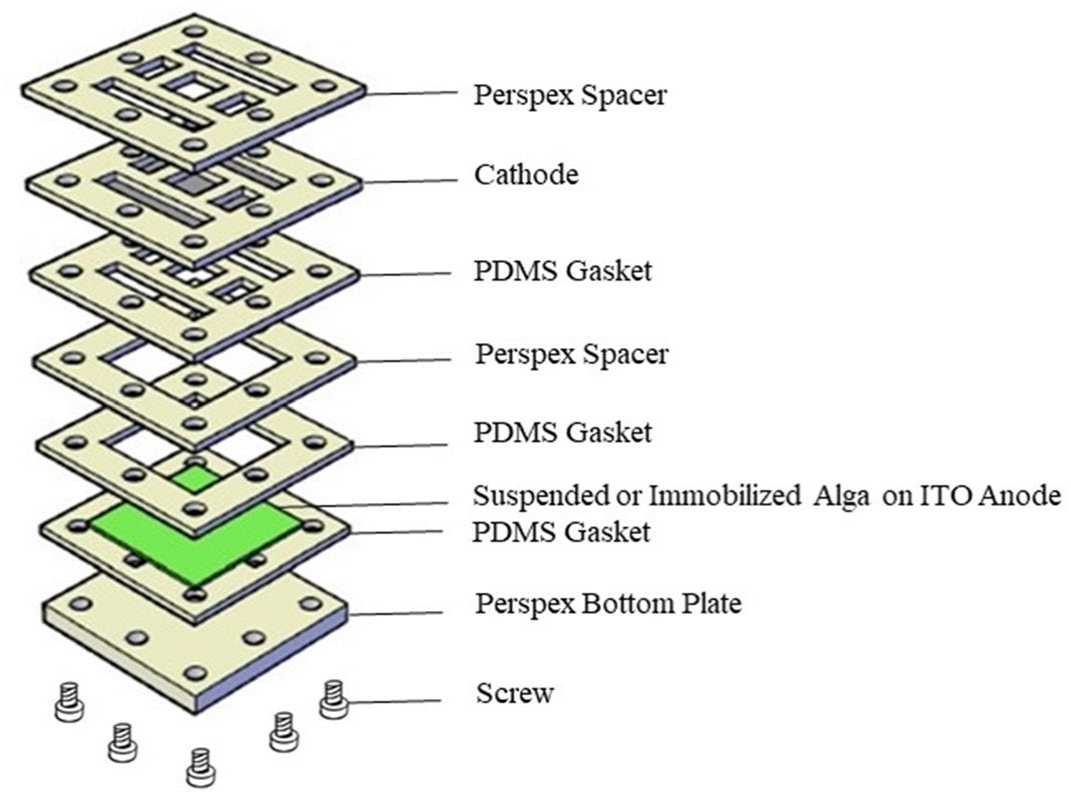
Exploded view of the ITO based BPV device.

The algal BPV device was a closed, single-chamber BPV, consisting of a 50 × 50 mm platinum-coated glass cathode, which was placed in parallel with an ITO coated glass (KINTEC, Hong Kong) anode. The body of the open*-*air, single-chamber BPV was constructed of clear Perspex (Fig. 8).

### Preparation of immobilized algae

Immobilized algae were grown on the surface of the anode in a clear Perspex chamber sealed with polydimethylsiloxane (PDMS) and then filled with fresh BBM. The algal cells were immobilized in 2% sodium alginate. Sodium alginate powder (2 g; Natural Colloids Industries Pte. Ltd.) was added into 95 mL of sterile distilled water and stirred continuously using a magnetic stirrer overnight to prepare the 2% sodium alginate solution. *Chlorella* sp. cells were suspended in BBM to form a concentrated algal suspension (OD_620nm_ = 2.0) of which 5 mL were added to the 95 mL sodium alginate solution to form the algal alginate suspension. ITO-coated 3.5 cm × 3.5 cm glass slides were placed in a mould made by sterile aluminium foil. A pipette was used to spread 3 mL of the algal alginate suspension on the ITO-coated glass slides and the algal alginate suspension allowed to settle for 15 min. Sterile CaCl_2_ (0.1 M) was sprayed onto the surface of the suspension until it was fully covered. Samples were left for one hour to complete the gelation process to form a 2 mm thick algal gel film on the ITO. The film was measured with Elcometer 3230 Wet Film Wheels. The ITO anode with the algal gel film was rinsed with sterile distilled water to remove the CaCl_2_ solution after the culture immobilization process was completed^6^.

### Preparation of suspended algae

The algal suspensions were prepared using exponential phase cultures in BBM, where 5 mL of concentrated *Chlorella* sp. (OD_620nm_ = 2.0) was added to the 95 mL BBM medium. Suspension cell cultures, for comparison with the immobilized cultures, were grown in the same way as above, and placed on top of the ITO anode in the BPVs.

### Chl*-a* and carotenoids concentration

Biomass was estimated based on chl*-a* content. Carotenoid concentration was used as a stress indicator^40^. The chl*-a* and carotenoid concentrations were determined using the spectrophotometric method^41^. The suspended culture was pipetted out from the BPV and collected on a glass-fibre filter paper (Whatman GF/C, 0.45 µm). For the immobilized algae, the alginate layer was removed from the ITO anode. Both types of samples were mashed in 10 mL of analytical grade 100% acetone using a hand homogenizer. The samples were then kept at 4 ºC for 24 h before centrifugation at 3000 rpm for 10 min at 4 ºC. Absorption of the supernatant was measured at 630 nm (OD_630_), 645 nm (OD_645_), 665 nm (OD_665_), and 452 nm (OD_452_), using a Shimadzu UV 1800 spectrophotometer. All measurements were conducted in triplicates. All statistical analyses were performed using the Statistica 8 program.

The chl*-a* concentration was calculated using the following formula:

$$ {\text{Chl-a}}\left( {{\text{mg m}}^{{ - {3}}} } \right) \, = \, \left( {{\text{Ca }} \times {\text{ Va}}} \right)/{\text{Vc}}$$

where

$$ {\text{Ca } = \text{ 11}}{.6} \times {\text{OD}}_{{{\text{665nm}}}} - 1.31 \times {\text{OD}}_{{{\text{645nm}}}} - 0.14 \times {\text{OD}}_{{{\text{630nm}}}} . $$

Va is the volume of acetone (mL) used for extraction, Vc is the volume of culture (L).

$$ {\text{Chl-a (mg/L) = Chl-a (mg m}}^{{{ - }{3}}} {)/1000}{\text{.}} $$

The carotenoid concentration was calculated using the following formula:

$$ {\text{Carotenoids (mg}}/{\text{L) }} = \, \left( {{\text{OD}}_{{{452}}} \times { 3}.{86 } \times {\text{ Va}}} \right)/{\text{Vc}} $$

where Va is the volume of acetone (mL) used for extraction, Vc is the volume of culture (mL).

### Cell count

Cell density (cells mL^−1^) was also used for biomass estimation and was determined using an improved Double-Neubauer Haemocytometer^42^. Appropriate dilution and homogenization of the algal cultures were performed to limit the cell number to below 100 per counting chamber.

### Specific growth rate

The specific growth rate (µ) was calculated based on chl*-a* content (mg L^−1^) using the following formula:

$${\text{SGR}}, \, \mu {\text{ (day}}^{{ - {1}}} {) } = \, \left( {{\text{Ln N}}_{{2}} - {\text{Ln N}}_{{1}} } \right)/{\text{ t}}_{{2}} - {\text{t}}_{{1}}$$

where N_2_ is the chl*-a* (mg L^−1^) at t_2_ time, N_1_ is the chl*-a* (mg L^−1^) at t_1_ time, t_2_ and t_1_ are the  times within the exponential phase^42^.

### Carbon and CO_2_ fixation

The carbon fixation and CO_2_ fixation rates were estimated as follows^43^:

$$ {\text{Carbon fixation}},{\text{ mg carbon L}}^{{ - {1}}} = { 5}0\% {\text{ of the cell'}} {\text{s biomass}} $$

where biomass (mg) = Chl-a content (mg L^−1^) × 67, assuming that Chl-a makes up 1.5% of the cell 478 biomass.

$$ {\text{CO}}_{2} \;{\text{fixation rates}},{\text{ mg L}}^{{ - 1}} \;{\text{day}}^{{ - 1}}  = 0.5P \times (44/12) $$

where P is the biomass productivity = (biomass at t_2_ – biomass at t_1_)/(t_2_ – t_1_), 44 is the molecular weight of carbon dioxide (gmol^−1^), 12 is the atomic weight of carbon (gmol^−1^)^43^.

### Pulse amplitude modulation fluorometer measurement

Fluorescence analysis was employed to measure the photosynthetic performance of algal cultures in the BPV devices. Photosynthetic parameters in this study were measured using a pulse amplitude modulation (PAM) fluorometer (Diving PAM, Walz, Germany)^44^. A rapid light curve (RLC) was obtained under software control (Wincontrol, Walz) using eight consecutive ten-second intervals of actinic light with increasing intensity (light levels: 0, 47, 139, 272, 414, 602, 817, 1179 and 1566 µmol photons m^−2^ s^−1^). The cultures were dark-adapted for 15 min before exposure to different light level. The maximum photosynthetic efficiency was determined from the initial slope (α) of the RLC. The relative electron transport rate (rETR) was calculated by multiplying the irradiance by the quantum yield (ratio of the number of photons emitted to the number of photons absorbed) measured with the Diving-PAM at the end of each light interval. The photoadaptive index (E_k_) is the value of the irradiance where the rETR is a maximum^45^. Maximum quantum efficiency (F_v_/F_m_), was used to indicate if the cells were stressed by the exposure to light: ${\text{F}}_{{\text{v}}} {\text{/F}}_{{\text{m}}} {\text{ = (F}}_{{\text{m}}} {\text{ } - \text{ F}}_{{0}} {\text{)/F}}_{{\text{m}}}$, where F_m_ is the maximum fluorescence and F_0_ is the minimum fluorescence.

### BPV electrical measurement

Crocodile clips and copper wire were used to connect the anode and cathode of the BPV devices to the external circuit. Polarization curves (voltage vs current density) were constructed by applying external resistance stepping technique, where the external resistance was decreased every 10 min with corresponding voltage measurement using a multimeter (Agilent U1251B). The current is derived using Ohm’s Law. Different range of external resistance were tested to find suitable resistance with respective of voltage drop across the resistor. The sequence of external resistance was 10 MΩ, 5.6 MΩ, 2 MΩ, 560 KΩ, 240 KΩ, 62 KΩ, 22 KΩ, 9.1 KΩ, 2.7 KΩ and 1.1 KΩ. The maximum current density and power density are evaluated from the polarization curve. Each measurement was repeated in triplicate. All statistical analyses were performed using the Statistica 8 program.

### Absorption spectrum of pigments from Chlorella UMACC 313—measured and simulated

Pigments (chlorophylls and carotenoids) were extracted from an exponential culture of *Chlorella* UMACC 313. The pigment extract (1 mL) was subjected to scanning from 350 to 700 nm at intervals of 20 nm, using the Shimadzu UV 1800 spectrophotometer, to generate the absorption spectrum. This measured absorption spectrum is simulated in the light provided by a PLA with 16 LEDs (Telelumen LLC, Saratoga, CA, US).
